# Maternal and offspring genetic risk score analyses of fetal alcohol exposure and attention‐deficit hyperactivity disorder risk in offspring

**DOI:** 10.1111/acer.14692

**Published:** 2021-09-06

**Authors:** Elis Haan, Hannah M. Sallis, Eivind Ystrom, Pål Rasmus Njølstad, Ole A. Andreassen, Ted Reichborn‐Kjennerud, Marcus R. Munafò, Alexandra Havdahl, Luisa Zuccolo

**Affiliations:** ^1^ School of Psychological Science University of Bristol Bristol UK; ^2^ MRC Integrative Epidemiology Unit University of Bristol Bristol UK; ^3^ Department of Psychology PROMENTA Research Center University of Oslo Oslo Norway; ^4^ Department of Mental Disorders Norwegian Institute of Public Health Oslo Norway; ^5^ Department of Clinical Science Center for Diabetes Research University of Bergen Bergen Norway; ^6^ Department of Pediatrics and Adolescents Haukeland University Hospital Bergen Norway; ^7^ NORMENT Centre Institute of Clinical Medicine University of Oslo Oslo Norway; ^8^ Division of Mental Health and Addiction Oslo University Hospital Oslo Norway; ^9^ Institute of Clinical Medicine University of Oslo Oslo Norway; ^10^ Nic Waals Institute Lovisenberg Diaconal Hospital Oslo Norway; ^11^ Department of Population Health Sciences Bristol Medical School University of Bristol Bristol UK

**Keywords:** ALSPAC, attention‐deficit hyperactivity disorder, fetal alcohol exposure, GenR, MoBa

## Abstract

**Background:**

Studies investigating the effects of prenatal alcohol exposure on childhood attention‐deficit hyperactivity disorder (ADHD) symptoms using conventional observational designs have reported inconsistent findings, which may be affected by unmeasured confounding and maternal and fetal ability to metabolize alcohol. We used genetic variants from the alcohol metabolizing genes, alcohol dehydrogenase (*ADH*) and aldehyde dehydrogenase (*ALDH*), as proxies for fetal alcohol exposure to investigate their association with risk of offspring ADHD symptoms around age 7–8 years.

**Methods:**

We used data from 3 longitudinal pregnancy cohorts: Avon Longitudinal Study of Parents and Children (ALSPAC), Generation R study (GenR), and the Norwegian Mother, Father and Child Cohort study (MoBa). Genetic risk scores (GRS) for alcohol use and metabolism using 36 single nucleotide polymorphisms (SNPs) from *ADH* and *ALDH* genes were calculated for mothers (*N*_ALSPAC_ = 8196; N_MOBA_ = 13,614), fathers (*N*_MOBA_ = 13,935), and offspring (*N*_ALSPAC_=8,237; *N*_MOBA_=14,112; *N*_GENR_=2,661). Associations between maternal GRS and offspring risk of ADHD symptoms were tested in the full sample to avoid collider bias. Offspring GRS analyses were stratified by maternal drinking status.

**Results:**

The pooled estimate in maternal GRS analyses adjusted for offspring GRS in ALSPAC and MoBa was OR = 0.99, 95%CI 0.97–1.02. The pooled estimate in offspring GRS analyses stratified by maternal drinking status across all the cohorts was as follows: OR_DRINKING_ = 0.98, 95% CI 0.94–1.02; OR_NO DRINKING_ = 0.99, 95% CI 0.97–1.02. These findings remained similar after accounting for maternal genotype data in ALSPAC and maternal and paternal genotype data in MoBa.

**Conclusions:**

We did not find evidence for a causal effect of fetal alcohol exposure on risk of ADHD symptoms in offspring. The results may be affected by limited power to detect small effects and outcome assessment.

## Graphical abstract

In this study, we use genetic variants in *ADH/ALDH* as proxies for fetal alcohol exposure and investigate their association with offspring ADHD risk, and separately with hyperactive‐impulsive and inattention symptom domains, using data from three large European birth cohorts: the Avon Longitudinal Study of Parents and Children (ALSPAC), Generation R (GenR) and the Norwegian Mother, Father and Child Cohort Study (MoBa). We did not find clear evidence for a causal effect of fetal alcohol exposure on ADHD risk in offspring.

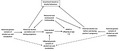

## INTRODUCTION

It is well documented that heavier alcohol consumption during pregnancy can negatively affect fetal development and later neurodevelopmental problems (Patra et al., 2011), but evidence is inconsistent regarding the effects of low prenatal alcohol exposure (PAE) (Mamluk et al., 2017). The harmful neurodevelopmental effects resulting from PAE are collectively defined as fetal alcohol spectrum disorders (FASD), which include fetal alcohol syndrome (FAS), partial FAS, alcohol‐related neurodevelopmental disorder (ARND), alcohol‐related birth defects (ARBD), and neurobehavioral disorder associated with prenatal alcohol exposure (ND‐PAE) (Mattson et al., 2019). However, there is substantial overlap between FASD, attention‐deficit hyperactivity disorder (ADHD), and other behavioral impairments (Popova et al., 2016; Weyrauch et al., 2017). A lack of clear diagnostic criteria of FASD makes it difficult to distinguish from other neurodevelopmental disorders (Burd, 2016; Lange et al., 2019). Although ADHD is one of the most common neurodevelopmental disorder diagnoses in FASD, little is known about the role of PAE in causing ADHD symptoms in the general population.

A systematic review by Easey and colleagues suggested that low and moderate alcohol consumption during pregnancy is associated with negative mental health outcomes in children, including anxiety/depression, total behavioral problems, and conduct disorder (Easey et al., 2019). However, another recent systematic review and meta‐analysis focusing specifically on offspring ADHD found little evidence to suggest an increased risk of ADHD symptoms in children whose mothers consumed moderate amounts of alcohol (up to 70 g a week) during pregnancy (Porter et al., 2019). In contrast, low PAE was found to have a protective effect on ADHD symptoms in some earlier studies (Kelly et al., 2013; Niclasen et al., 2014). However, it is possible that these associations are due to genetic confounding or confounding by social factors, as both studies found that women who drank low or moderate levels during pregnancy had a higher socioeconomic position, which is negatively associated with ADHD risk in the offspring. Furthermore, studies based on siblings discordant on PAE that account for shared genetics can strengthen evidence for causality. In these studies, PAE showed a minor residual effect on internalizing problems up to age 3 (Lund et al., 2019) and ADHD symptoms at 5 years (Eilertsen et al., 2017). Most of the effect was accounted for by maternal factors shared by siblings. In these studies, bias because of unmeasured confounding and nonshared environmental factors cannot be ruled out.

One potential approach to overcome limitations due to unmeasured confounding is to use genetic variants predictive of alcohol consumption or directly involved in alcohol metabolism to disentangle effects of PAE on child outcomes. Using a Mendelian Randomization (MR) design can also help to investigate causality, as the genetic variants used as a proxy for the exposure are generally less affected by confounding factors than the self‐reported exposure measurements used in more conventional epidemiological analyses (Davey Smith & Hemani, 2014). If we observe evidence of an association between the genetic proxy and the outcome, this provides stronger support for a causal influence of the exposure (prenatal alcohol consumption) on our outcome (risk of offspring ADHD symptoms) (Davey Smith & Ebrahim, 2003).

Our recent report using a combination of negative control and polygenic risk score (PRS) analyses did not find evidence for a causal effect of PAE on offspring ADHD symptoms risk (Haan et al., 2021). In this previous study, the alcohol PRS was derived using SNPs identified from the latest GWAS on alcohol consumption per week (Liu et al., 2019). However, the alcohol PRS was based on a discovery sample of general population individuals, who on average consume more alcohol than pregnant women. Moreover, there is evidence that harmful effects of alcohol exposure are also affected by maternal and fetal metabolic capacity, which can lead to different fetal alcohol levels if pregnant women drink alcohol (Burd et al., 2012). Therefore, analyses based on the PRS of alcohol consumption per week could miss biologically important effects related to individual differences in the ability to metabolize alcohol. A large body of research has shown that ethanol metabolism is affected by a group of alcohol dehydrogenase (*ADH*: *ADH1A*, *ADH1B*, *ADH1C*, *ADH4*, *ADH5*, *ADH6*, *ADH7*) and aldehyde dehydrogenase (*ALDH*: *ALDH1A1* and *ALDH2*) genes, and variants in these genes impact how quickly alcohol is metabolized, the amount of alcohol consumed and individual effects of alcohol consumption (Birley et al., 2009; Birley et al., 2008; Edenberg & McClintick, 2018). Furthermore, genetic variants of ADH genes are also expressed in early fetal development. After maternal alcohol intake, fetal blood alcohol concentration is nearly equivalent to maternal alcohol levels (Burd et al., 2012) and although the fetus can metabolize some alcohol, the majority of alcohol metabolism acts through maternal metabolic pathways (Burd et al., 2012). The importance of fetal ADH genetic variants is evidenced by previous studies using the ALSPAC sample, which have shown that 4 child *ADH* genetic variants were associated with lower IQ at age 8 and early onset conduct problems in children whose mothers drank during pregnancy (Lewis et al., 2012; Murray et al., 2016). However, these studies used only a limited number of *ADH* genetic variants selected based on the association with the outcome, and it is not fully clear how these 4 genetic variants change metabolic activity.

**FIGURE 1:**
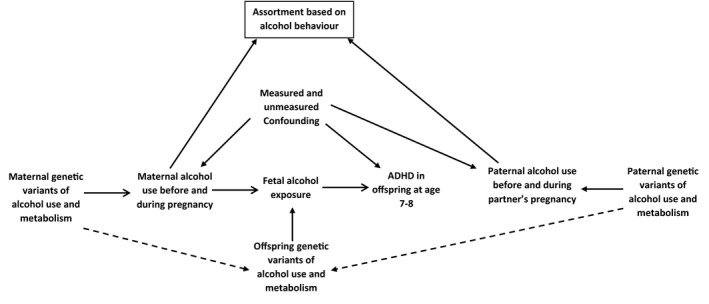
Study design: maternal and offspring genetic variants of alcohol use and metabolism were used as proxies for fetal alcohol exposure (the exposure of interest, unmeasured) to investigate associations with ADHD risk in offspring around age 7–8 years (outcome). Dashed arrows represent genetic correlation as a child inherits 50% of its genetic makeup from mother and father. In this study, we assume that mate selection is not based on confounders but could be affected by alcohol behavior. Adjustment to paternal genetic data will help to overcome potential bias because of the assortment and shared genetics

It remains unclear whether PAE has a causal effect on risk of offspring ADHD symptoms, through modulations of maternal and fetal alcohol metabolism. In this study, we use genetic variants in *ADH*/*ALDH* as proxies for fetal alcohol exposure and investigate their association with risk of offspring ADHD symptoms, and separately with hyperactive‐impulsive and inattention symptom domains, using data from 3 large European birth cohorts (Figure 1).

## MATERIALS AND METHODS

### Study populations

We used data from 3 European prospective longitudinal birth cohorts: the Avon Longitudinal Study of Parents and Children (ALSPAC), Generation R (GenR) and the Norwegian Mother, Father and Child Cohort Study (MoBa). ALSPAC is a prospective longitudinal cohort study that recruited 14,541 pregnant women resident in Avon, UK, with expected dates of delivery between April 1, 1991, and December 31, 1992 (Boyd et al., 2013; Fraser et al., 2013; Northstone et al., 2019). Generation R (GenR) is a population‐based prospective cohort study in Rotterdam in the Netherlands that recruited 9,778 pregnant women expected to give birth between April 2002 and January 2006 (Kooijman et al., 2016). MoBa is a population‐based pregnancy cohort study where participants were recruited from all over Norway between 1999 and 2008. The cohort now includes 114,500 children, 95,200 mothers and 75,200 fathers (Magnus et al., 2016). More details are shown in the Supplementary Material.

### Availability of genome‐wide data

In ALSPAC, genome‐wide data are available for 8,237 children and 8,196 mothers. In GenR, genetic data are available for 2,661 children from European ancestry, but maternal genetic data were not available at the time of analyses. In MoBa, genetic data were available for 14,112 children, 13,614 mothers, and 13,935 fathers. Detailed information about the genotyping is presented in the Supplementary Material.

### Exposures

In ALSPAC, at 18 weeks’ gestation mothers were asked about their average amount and frequency of alcohol consumption (a) during the first trimester, (b) in the previous 2 weeks, and (c) at the time when they first felt the baby move. At 32 weeks’ gestation, mothers were asked about their average weekday and weekend alcohol consumption.

In GenR, in the first questionnaire (<18 weeks) mothers were asked whether they drank any alcohol in the first 3 months of pregnancy. In mid‐pregnancy (18–25 weeks) and late pregnancy (>25 weeks), mothers were asked if they drank alcohol in the past 2 months and mothers who reported drinking were asked to classify their average alcohol consumption.

In MoBa, at 15 and 30 weeks’ gestation mothers were asked how often and how many units of alcohol they consumed in the current pregnancy. Six months after their child's birth, mothers were asked about their alcohol consumption (amount and frequency) during the last 3 months of their pregnancy.

Mothers who reported alcohol consumption at any point during the pregnancy were classified as drinkers. More details are presented in Table S1.

### Outcome

ADHD symptoms were measured around age 7–8 years in each cohort. As in our previous study (Haan et al., 2021), we wanted to explore whether we observe differences in ADHD hyperactive‐impulsive and inattention symptom domains, if ADHD symptoms were reported by mother or teacher, and whether results depended on the questionnaire used for ADHD assessment.

As the continuous score of ADHD symptoms was either zero‐inflated or skewed, a binary variable was derived for total ADHD, hyperactive‐impulsive, and inattention symptoms using the 85^th^ percentile as a threshold to indicate the presence of high‐level symptoms (Achenbach & Rescorla, 2001; Aylward & Stancin, 2008). Up to 4 missing items were allowed depending on the total number of items in the questionnaire. More details can be found in Table S2.

#### *Primary* *outcome measure*

The psychometric scales used for the main outcome measure were as follows: maternal report of the Development And Well‐Being Assessment (DAWBA) questionnaire in ALSPAC; maternal report of the revised Conner's Parent Rating Scale (CPRS‐R) in GenR; and maternal report of the Disruptive Behaviour Disorders scale (RS‐DBD) in MoBa.

#### *Secondary* *outcome measures*

We additionally included teacher report of the DAWBA questionnaire and maternal and teacher report of the Strengths and Difficulties Questionnaire (SDQ) hyperactivity subscale in ALSPAC, and maternal and teacher report of the Child Behaviour Checklist (CBCL) attention problems subscale in GenR.

### Genetic variants

Based on previous research, we identified genes responsible for alcohol metabolism that were expressed in liver and brain using Expression Atlas (Kapushesky et al., 2010; Papatheodorou et al., 2020). Using this online tool, we identified *ADH1A*, *ADH1B*, *ADH4*, *ADH5*, *ADH6*, *ALDH2*, *ALDH1A1,* and *ALDH1B1* that were expressed in adults and fetus, and additionally *ADH1C* and *ADH7* genes, which were only expressed in adults. All these genes are located in chromosomes 4, 9, and 12. In total, 869 single nucleotide polymorphisms (SNPs) from these genes were identified in ALSPAC (Figures S1–S3). Given the high linkage disequilibrium of these SNPs, we used a clumping procedure (*R* ^2^ < 0.01) to identify independent SNPs. 1000 genomes were used as a reference panel for clumping. After clumping, we identified 36 independent SNPs (Table S3).

### Harmonization

Before calculating genetic risk scores (GRS), SNPs were aligned so that the effect alleles were positively associated with alcohol consumption in the discovery sample. These effect estimates were taken from the summary statistics of the latest GWAS on alcohol consumption per week (Liu et al., 2019). The harmonization procedure in each cohort is shown in Tables S4–S6. All SNPs identified in ALSPAC were also available in GenR. In MoBa, genetic imputation was conducted using the Haplotype Reference Consortium (HRC) as a reference panel, and 9 SNPs identified in ALSPAC were unavailable in MoBa. We identified proxies for these SNPs using Single Nucleotide Polymorphisms Annotator (SNiPA) (Arnold et al., 2015) (Table S6).

### Genetic risk scores

As it is not possible to directly measure the effect of each SNP on fetal exposure to alcohol, and therefore to calculate weights, we derived unweighted offspring and parental GRS using PLINK 1.9. Generation R data only allowed computation of offspring GRS, ALSPAC allowed computation of offspring and maternal GRS, and MoBa allowed computation of offspring, maternal, and paternal GRS.

### Statistical analyses

All analyses were performed using Stata (v15: ALSPAC, GenR; v16: MoBa) (StataCorp, 2019; StataCorp, 2017) and restricted to unrelated individuals of European ancestry. Analyses were performed as described in our preregistered protocol (Haan et al., 2019). Associations between maternal and offspring GRS and risk of ADHD symptoms in offspring were tested using logistic regression. All analyses were adjusted for 10 ancestry informed principal components and in MoBa additionally for birth year and genotyping batch. Thanks to the harmonization procedure described above, estimates of association between GRS and ADHD risk can be interpreted as odds ratios (ORs) per additional alcohol‐increasing GRS allele—a positive OR likely indicates a detrimental effect of fetal alcohol exposure on risk of ADHD symptoms.

### Primary analysis

We carried out analyses using both maternal and offspring GRS as proxies for increased fetal alcohol exposure. Maternal analyses were performed using 3 models: (1) maternal GRS; (2) maternal GRS adjusted for offspring GRS; (3) maternal GRS adjusted for offspring and paternal GRS. These analyses were performed in the full sample without stratifying based on maternal drinking status, because this could induce collider bias as genetic propensities of women who drink during pregnancy may differ from those women who abstain, thus introducing a spurious association between the GRS and confounders (Figure S4a). Three models were used also in offspring GRS analyses: (1) offspring GRS; (2) offspring GRS adjusted for maternal GRS; (3) offspring GRS adjusted for maternal and paternal GRS. These analyses were stratified by maternal drinking status during pregnancy, since because collider bias is not an issue with respect to fetal alcohol exposure (Figure S4b). Additionally, in model 3, where maternal GRS analyses were adjusted for offspring and paternal GRS in MoBa, we were able to account for any shared genetic component that might confound the maternal alcohol GRS–offspring ADHD association.

Analyses were performed separately in each cohort and then meta‐analyzed across the cohorts using a random effects model, which accounts for variability in the exposure and outcome assessment across the cohorts. ALSPAC and MoBa results from model 2 (maternal GRS adjusted for offspring GRS) were pooled in meta‐analyses, while all 3 cohorts contributed to meta‐analyses for the offspring GRS model 1 results.

### Sensitivity analyses

We also checked the influence of each individual SNP on the outcome using a leave‐one‐out approach. We created 36 additional GRS for mothers and offspring excluding one SNP at a time. Given that there was no deviation in effect estimates when leaving out individual SNPs (Figures S5–15), we used the GRS including all 36 SNPs in our analyses. We also tested the associations between maternal GRS and potential confounders.

### Replication analyses of previous ALSPAC studies

We also created a GRS including 4 offspring *ADH* genetic variants (*ADH1A*: rs975833, rs2866151; *ADH1B*: rs4147536; *ADH7* rs284779) found to be associated with neurodevelopmental outcomes in previous ALSPAC studies (Lewis et al., 2012; Murray et al., 2016).

## RESULTS

An overview of study sample characteristics is shown in Table 1. In all the cohorts, mothers who reported drinking were older and better educated, compared to nondrinking mothers.

**TABLE 1:** Study sample characteristics

Confounders	ALSPAC		GenR		MoBa	
	Maternal alcohol consumption during pregnancy					
	No *N* = 1,125	Yes *N* = 2,573	No *N* = 1,004	Yes *N* = 1,659	No *N* = 6,536	Yes *N* = 1,357
Mother's age in years (mean and SD)	28 (4.5)	30 (4.3)	30 (4.8)	32 (4.1)	30 (4.3)	32 (3.9)
Mother's education						
Primary	14%	8%	10%	2%	1%	1%
Secondary	71%	69%	49%	28%	27%	19%
Higher	15%	23%	41%	70%	72%	80%
Financial difficulties^a^						
No	42%	40%	80%	90%	87%	86%
Yes	58%	60%	20%	10%	13%	14%
Marital status						
Married	84%	85%	92%	92%	98.5%	98%
Single/not married	16%	15%	8%	8%	1.5%	2%
Depression symptoms^b^	9%	10%	8%	5%	5%	6%
Anxiety symptoms	12%	14%	10%	6%		
Mothers ADHD symptoms					2%	3%
Parity						
1st	53%	45%	57%	61%	51%	40%
2nd	33%	37%	30%	30%	33%	39%
3rd+	14%	18%	13%	9%	16%	21%
Mother smoked during pregnancy	15%	17%	17%	25%	5%	7%
Child ADHD symptoms above the 85th percentile threshold	13%	14%	13%	15%	12%	12%

^a^ In ALSPAC, financial difficulties were measured with 5 items questionnaire: (1) Difficulty in affording food; (2) Difficulty in affording clothing; (3) Difficulty in affording heating; (4) Difficulty in affording accommodation; (5) Difficulty in affording things for baby. In GenR, financial difficulties were assessed with single item question: Difficulty in paying food, rent, bills, and suchlike. In MoBa, financial difficulties were assessed with single item question: Have you experienced financial problems?

^b^ In MoBa, depression and anxiety problems were assessed together.

### Maternal GRS analysis

**FIGURE 2:**
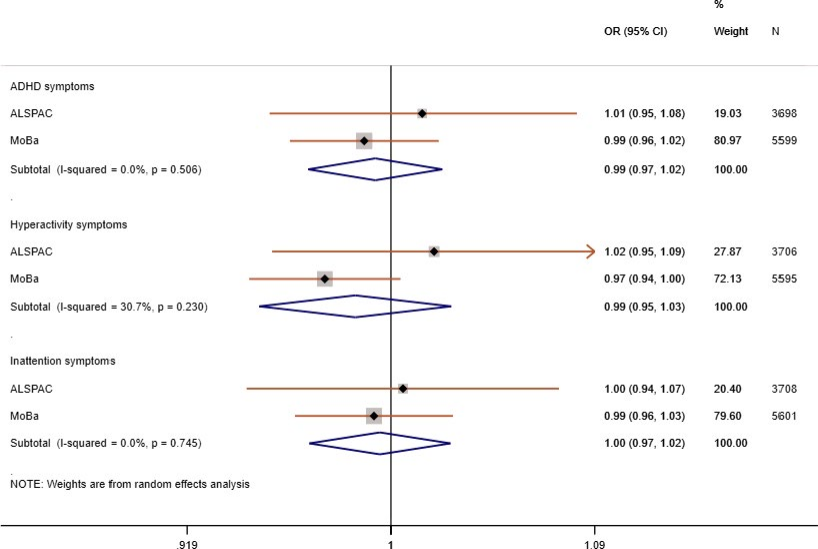
Meta‐analysis of maternal GRS on high risk of maternal reported offspring ADHD symptoms in ALSPAC and MoBa: Model 2—maternal GRS adjusted for offspring GRS and 10 ancestry principal components

**FIGURE 3:**
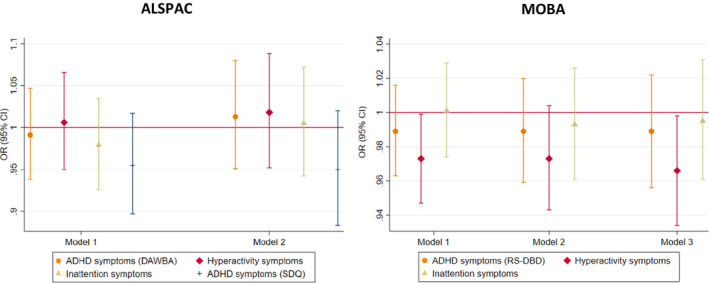
Associations between maternal GRS and high risk of maternal reported offspring ADHD symptoms in ALSPAC and MoBa: Model 1—only maternal GRS; Model 2—maternal GRS adjusted for offspring GRS; Model 3—maternal GRS adjusted for offspring and paternal GRS; all analyses are adjusted for 10 ancestry principal components. In MoBa, also for birth year and genotyping batch; Development And Well‐Being Assessment (DAWBA); Strengths and Difficulties Questionnaire (SDQ)—secondary measure; Rating Scale for Disruptive Behavior Disorders (RS‐DBD)

The pooled estimate for the association of maternal GRS with high risk of maternal reported ADHD symptoms in offspring (model 2—adjusted for offspring GRS) did not show evidence for an association (OR_ADHD_ = 0.99, 95% CI 0.97–1.02; OR_HYP_ = 0.99, 95% CI 0.95–1.03; OR_INA_ = 1.00, 95% CI 0.97–1.02) (Figure 2). In MoBa, we found weak evidence of a negative association between maternal GRS and high risk of maternal reported offspring symptoms in the hyperactivity domain only, in model 1 (maternal GRS) and model 3 (adjusted for offspring and paternal GRS) (Figure 3, Table S7). This finding was not replicated in ALSPAC using either maternal or teacher report (Figure 3, Figure S16, Tables S8 & S9).

### Offspring GRS analysis

**FIGURE 4:**
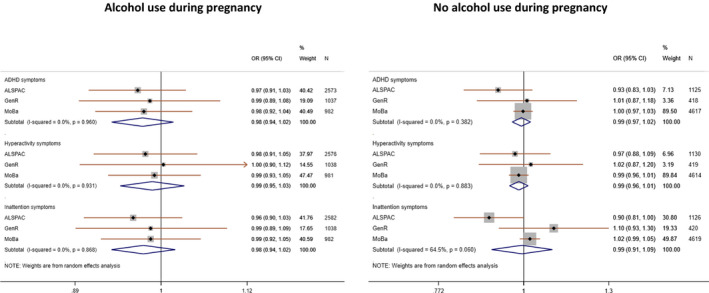
Meta‐analysis of offspring GRS on high risk of maternal reported offspring ADHD symptoms across the cohorts: Model 1—only offspring GRS and adjusted for 10 ancestry principal components

**FIGURE 5:**
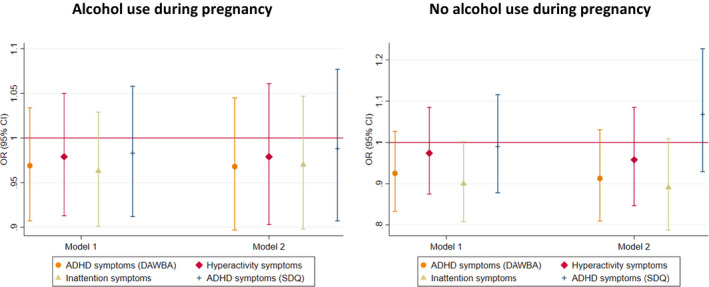
Associations between offspring GRS and high risk of maternal reported offspring ADHD symptoms in ALSPAC stratified by maternal drinking status in ALSPAC: Model 1—only offspring GRS; Model 2—offspring GRS adjusted for maternal GRS; all analyses are adjusted for 10 ancestry principal components; Development And Well‐Being Assessment (DAWBA); Strengths and Difficulties Questionnaire (SDQ)—secondary measure

**FIGURE 6:**
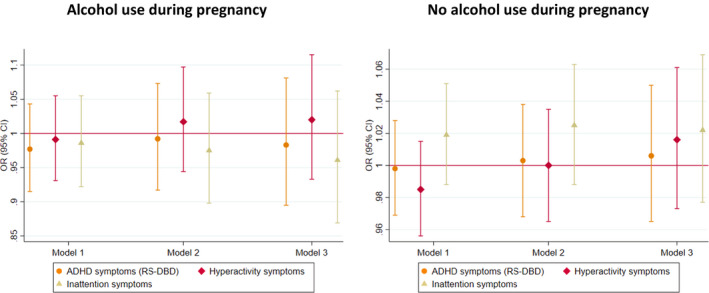
Associations between offspring GRS and high risk of maternal reported offspring ADHD symptoms in MoBa stratified by maternal drinking status in MoBa: Model 1—only offspring GRS; Model 2—offspring GRS adjusted for maternal GRS; Model 3—offspring GRS adjusted for maternal and paternal GRS; all analyses are adjusted for 10 ancestry principal components, birth year and genotyping batch; Rating Scale for Disruptive Behavior Disorders (RS‐DBD)

**FIGURE 7:**
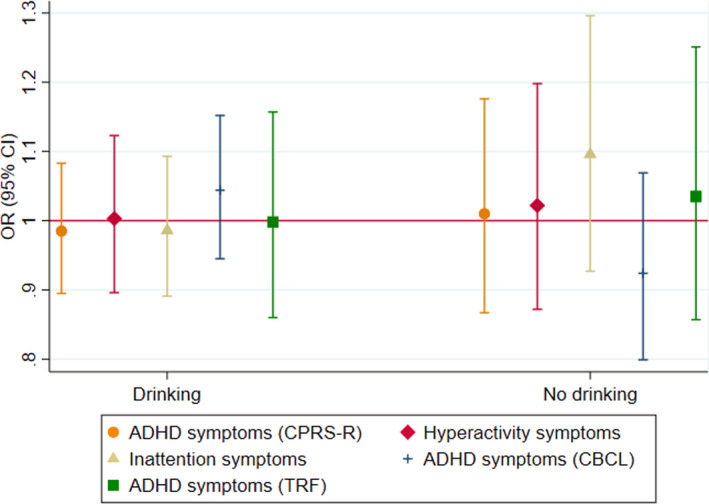
Associations between offspring GRS and high risk of maternal and teacher reported offspring ADHD symptoms in GenR stratified by maternal drinking status: Model 1—only offspring GRS adjusted for 10 ancestry principal components; Revised Conner's Parent Rating Scale (CPRS‐R); Child Behavior Checklist (CBCL)—secondary measure; Teacher Report Form (TRF)—secondary measure

The pooled estimate of the association between offspring GRS and maternal reported risk of ADHD symptoms in offspring using model 1 (offspring GRS) found no strong evidence of an effect. This remained consistent regardless of drinking status during pregnancy (Drinking: OR_ADHD_ = 0.98, 95% CI 0.94–1.02; OR_HYP_ = 0.99, 95% CI 0.95–1.03; OR_INA_ = 0.98, 95% CI 0.94–1.02; No drinking: OR_ADHD_ = 0.99, 95% CI 0.97–1.02; OR_HYP_ = 0.99, 95% CI 0.96–1.01; OR_INA_ = 0.99, 95% CI 0.91–1.09) (Figure 4). These results did not change after adjusting for maternal GRS (model 2) or maternal and paternal GRS (model 3) in ALSPAC and MoBa (Figures 5 and 6, Tables S10‐S13). Similarly, when using secondary outcome measures in GenR and ALSPAC, we found no strong evidence of an association between offspring GRS and risk of ADHD symptoms in offspring (Figure 7, Figure S17, Tables S14‐S16).

### Sensitivity analysis

Sensitivity analyses testing the associations between maternal GRS and confounders found evidence for an association with higher likelihood of not being married in MoBa, but no clear evidence for associations were observed in ALSPAC (Table S17‐S18).

### Replication analysis of previous ALSPAC studies

Replication analysis using offspring GRS with 4 *ADH* SNPs were consistent with the main analysis and provided little evidence for an association between fetal alcohol exposure and high risk of maternal or teacher reported ADHD symptoms in offspring (Figures S18‐S21 and Tables S19‐S25).

## DISCUSSION

We used maternal and offspring variants in the *ADH* and *ALDH* genes linked to alcohol use and metabolism to investigate whether there is a causal effect of fetal alcohol exposure on risk of childhood ADHD symptoms. Consistent with previous work, we did not find evidence for a causal effect (Haan et al., 2021). We observed only weak evidence in one cohort (MoBa) of a negative association between maternal GRS and maternal reported offspring symptoms, in the hyperactivity domain only. However, this was not replicated in ALSPAC where mothers had more prevalent daily alcohol use in pregnancy (16% in ALSPAC and 0.45% in MoBa). We did not observe associations of offspring GRS for alcohol use and metabolism with high risk of maternal or teacher reported childhood ADHD symptoms, in any of the cohorts, or for any of the ADHD symptom domains, even after adjustment for maternal and paternal GRS where this was available.

Our findings are somewhat dissimilar from previous findings in ALSPAC, which found the risk of early‐onset conduct problems and decreased IQ scores to be associated with the *ADH* variants, particularly in children whose mothers drank during pregnancy (Lewis et al., 2012; Murray et al., 2016). However, these studies used a limited number of offspring genetic variants in *ADH* genes as a proxy for fetal alcohol exposure. The present study was based on larger numbers of mother–offspring dyads (4x more) and used all available *ADH*/*ALDH* variants. Other studies that have examined the association between maternal PAE and behavioral problems in offspring using quasi‐experimental designs (such as sibling comparison) to account for shared genetic and environmental factors found evidence for a potential causal effect on conduct disorder symptoms in childhood, and emotional reactiveness at age 3 (reduced aggressiveness at age 5) but not with ADHD symptoms (D'Onofrio et al., 2007; Lund et al., 2019). However, another study using a similar design in the MoBa cohort observed an association with ADHD symptoms at age 5 years measured with CPRS‐R, but not with CBCL or when ADHD diagnosis was used (Eilertsen et al., 2017). However, in studies that used a sibling comparison design, the association may be still affected by nonshared environmental confounders. The genetic variants used in our study can help to overcome the limitation of unmeasured confounding. Triangulating findings across the studies based on different study designs, which have different sources of bias, can provide a clearer indication of whether a causal relationship exists. Given that the results are not consistent across the studies, no strong conclusions can be made.

A common limitation of previous studies reporting a positive association between PAE, conduct disorder, and ADHD symptoms (e.g., Murray et al., (2016), Lund et al., (2019), D’Onofrio et al., (2007) and Eilertsen et al., (2017)) is that they were based on outcomes that were measured only using maternal report. We have previously found inconsistent results for a potential causal effect when offspring ADHD symptoms were reported by mothers compared to teachers (Haan et al., 2021). This is not unexpected, as several studies have previously shown informant discrepancies in the assessment of children's mental health problems, which may be affected by factors such as mothers’ own mental health and socioeconomic status (Collishaw et al., 2009; De Los Reyes & Kazdin, 2005). Differences in the findings between previous studies and our study may be also related to scales used for ADHD symptom assessment as observed in our previous study (Haan et al., 2021) and the study by Eilertsen et al., (2017). The child's age at the time of assessment and severity of the ADHD symptoms may as well have an impact on the observed results. It has been shown that presentation and manifestation of ADHD and other behavioral symptoms and diagnosis change from preschool to adolescence (Bunte et al., 2014; Curchack‐Lichtin et al., 2014; Martel et al., 2017). This may lead to different findings across development as ADHD symptoms have been found to decline with age (Faraone et al., 2006). It is therefore possible that observed associations may be influenced by reporter bias, child age, and scales used for ADHD assessment and methods that did not sufficiently account for unmeasured confounds. Considering that no other studies have used the same SNPs and outcome combination as the current study, it was not possible to meta‐analyze our findings with other previously reported results.

The major strength of this study is that it used a more comprehensive approach for identifying genetic variants affecting alcohol metabolism. We also compared findings across three international longitudinal cohorts of which one cohort includes genetic data from both parents and offspring. Having trio genetic data enables us to properly account for shared genetics and overcome potential biases such as dynastic effects and assortative mating (Davies et al., 2019).

However, several limitations should also be considered. First, our sample size may have been insufficient to detect a small effect of fetal alcohol exposure on offspring risk of ADHD symptoms. However, based on the confidence intervals we can rule out a large effect with a reasonable degree of confidence. MR studies require large sample sizes that can be difficult to gain in the context of intergenerational research and particularly in pregnancy substance use exposures, as many women reduce/stop using substances when planning pregnancy and while being pregnant. Second, although MR and GRS analyses are less affected by confounding, there are 3 assumptions that need to be met: (1) Genetic variants are associated with the exposure; (2) genetic variants are not associated with the confounders; (3) genetic variants are not independently associated with the outcome. We could not test the assumption 1 directly because it is not possible to directly measure the level of alcohol the fetus is exposed to. Furthermore, assumptions 2 and 3 cannot be formally verified. Therefore, the risk of horizontal pleiotropy (violation of assumption 3) where the genetic variant directly affects the outcome independently of the exposure of interest still remains. Third, it is not possible to measure fetal blood alcohol levels to examine whether a dose–response relationship exists. Fourth, due to the low minor allele frequency and clumping procedure our GRS did not include a genetic variant—rs1229984—in the *ADH1B* gene that has been found to have a functional role in alcohol metabolism (Zuccolo et al., 2009). The role of rs1229984 could be investigated more thoroughly in future studies. However, our study captures its effect to some degree as it is correlated with a nearby variant that we did include (rs141973904; *R* ^2^ = 0.55). Fifth, maternal PAE was based on self‐reports and mothers may have underreported their prenatal alcohol use due to social desirability bias. This may have caused bias in the offspring GRS analyses, which were stratified based on maternal drinking status during pregnancy. However, studies that have compared agreement of maternal self‐reported alcohol consumption and ethanol biomarkers have shown that self‐reports are more biased when information about prenatal alcohol consumption is asked retrospectively rather than prospectively (Eichler et al., 2016). In all of our cohorts, prenatal alcohol use was assessed prospectively. In addition, studies examining reliability of maternal self‐reported prenatal smoking by using a measure of cotinine have shown that in ALSPAC and MoBa, cotinine levels were higher in women who smoked during pregnancy compared to nonsmokers (Kvalvik et al., 2012; Taylor et al., 2014), indicating that self‐reported smoking is a valid marker for prenatal smoking exposure. Given the more widely recognized harms of prenatal smoking compared to prenatal alcohol use (especially at the time of enrollment), this offers further reassurance. Sixth, it is possible that lack of effects could be also influenced by dichotomizing outcome measures. Very often, dichotomized measures are used as the scales used in ADHD assessment (SDQ, CBCL, CPRS‐R) generate skewed distributions (Swanson et al., 2012). Considering that research suggests that ADHD symptoms across a population are continuously distributed (Ahmad & Hinshaw, 2017; Larsson et al., 2012), continuous measures associated with ADHD symptoms could be more sensitive measures to use for investigating effects of fetal alcohol exposure. Seventh, longitudinal birth cohorts may be affected by selection bias if some groups are underrepresented in the initial recruitment. Attrition over time can also affect representativeness, which may cause biased exposure–outcome associations (Munafò et al., 2018). For example, in MoBa women who smoked during pregnancy, lived alone, and had two previous births were underrepresented (Nilsen et al., 2009) and in GenR women who continued to participate in the study were older and better educated (Kooijman et al., 2016). In addition, analyses restricted to live births such as the present one may incur some degree of selection bias, in the form of collider bias (Liew et al., 2015), although it has been found that live birth bias is unlikely to affect studies investigating perinatal factors (Heinke et al., 2020).

## CONCLUSIONS

We did not find evidence for a causal effect of fetal alcohol exposure on offspring ADHD risk in the general population. Considering that previous studies have found that PAE can affect a wide range of neurodevelopmental domains (Mattson et al., 2019), future studies should use a multidimensional approach and measure multiple neurodevelopmental outcomes at the same time. It has been also suggested that combining genetic and epigenetic data would make it possible to better distinguish FASD profile (Lange et al., 2019). In addition, larger samples with trio genetic data would increase power to detect a potential causal effect of maternal environmental exposures (including fetal alcohol exposure) on offspring outcomes. Furthermore, studies in East Asian populations could hold more promise to detect a potential causal effect given the considerable evidence of larger effects of specific genetic variants on alcohol metabolism and consumption in these populations (Hurley & Edenberg, 2012; Quertemont, 2004).

## AUTHOR CONTRIBUTORS

All authors have contributed to the study. EH conducted the analysis and drafted the manuscript with input from all co‐authors. All authors have approved the final version of the manuscript for submission.

## RESEARCH ETHICS

The ALSPAC study was approved by the ALSPAC Ethics and Law Committee and the Local Research Ethics Committees and informed consent for the use of data collected via questionnaires and clinics was obtained from participants. The GenR study was approved by the local Medical Ethical Committee (MEC 198.782/2001/31). Written informed consent was obtained from all participating women. The establishment of MoBa and initial data collection was based on a license from the Norwegian Data Protection Agency and approval from The Regional Committees for Medical and Health Research Ethics. The MoBa cohort is now based on regulations related to the Norwegian Health Registry Act. The MoBa analyses were approved by The Regional Committees for Medical and Health Research Ethics (2016/1702).

## CONFLICT OF INTEREST

The authors have no conflict declared.

## Supporting information

Supplementary Material
